# Recovery rate and predictors among children aged 6–59 months with severe acute malnutrition in Addis Ababa, Ethiopia: a retrospective follow-up study

**DOI:** 10.3389/fped.2024.1348378

**Published:** 2024-10-25

**Authors:** Genanew Kassie Getahun, Wondimu Ayele Andabo, Abraham Shewamare, Molla Yigzaw Birhanu

**Affiliations:** ^1^ Department of Public Health, Menelik II Medical and Health Science College, Addis Ababa, Ethiopia; ^2^ Department of Public Health, Addis Ababa Medical and Business College, Addis Ababa, Ethiopia; ^3^ Department of Public Health, Debre Markos University, Debre Markos, Ethiopia

**Keywords:** retrospective cohort, predictors, recovery, severe acute malnutrition, Ethiopia

## Abstract

**Introduction:**

Severe acute malnutrition (SAM) is a critical public health concern in Ethiopia, contributing to high morbidity and mortality rates among children. Despite the improvement in hospital coverage and the development of standardized WHO treatment guidelines, recent reviews indicated a wide range in recovery rates (34%–88%) due to several context-specific factors. Understanding the factors influencing the recovery time can help inform targeted interventions and improve the overall management of SAM cases. Therefore, this study aimed to assess the time to recovery and predictors of children aged 6–59 months with severe acute malnutrition in Addis Ababa, Ethiopia, in 2023.

**Methods:**

An institutional-based retrospective follow-up study was conducted among children aged 6–59 months who were admitted to Tirunesh Beijing Hospital (TBH), Addis Ababa, Ethiopia, from July 2019 to June 2023. The Kaplan–Meir estimate and survival curve were used to compare the time to recovery using a log-rank test among different characteristics. A Cox proportional hazard regression analysis model was used to identify significant predictors of time to recovery. Finally, a *p*-value of <0.05 was used to declare a significant association.

**Results:**

The median survival time to recovery was 17 (95% CI: 16.39–17.60) days, and the incidence rate of recovery from SAM was 37.8 per 1,000 child days. Moreover, exclusive breastfeeding [adjusted hazard ratio (aHR): 1.97, 95% CI: 1.45–2.68], amoxicillin provision (aHR = 1.62, 95% CI: 1.11–2.35), and deworming (aHR = 2.14, 95% CI: 1.48–3.09) were protective factors. However, complications at admission (aHR = 0.41, 95% CI: 0.23–0.73) and diarrhea during admission (aHR = 0.64, 95% CI: 0.45–0.91) were identified as risk factors of recovery from SAM.

**Conclusion:**

The time to recovery among the current study participants was low compared with the sphere standard. Besides, exclusive breastfeeding, complications at admission, diarrhea, amoxicillin provision, and deworming were independent predictors. Therefore, appropriate provision of routine medication and early management of medical comorbidity as per the national SAM management protocol can reduce the mortality of children with severe acute malnutrition significantly.

## 1 Introduction

Severe acute malnutrition (SAM) among children aged 6–59 remains a large-scale problem and a significant concern for most developing countries (1, 2). Globally, around 45%–60% of deaths for children aged less than 5 years were linked to undernutrition, and this is mostly occurring in low- and middle-income countries such as Ethiopia (2–4).

The current global burden of malnutrition is unacceptably high, and it affects every country on the planet Earth (5). In 2019, 144 million under-five children were stunted, 47 million were wasted, and 14.3 million were severely wasted worldwide (6). More than 90% of the burden was in Africa and southeast Asia (7). Despite continued prevention efforts, severe acute malnutrition remains a major public health problem in sub-Saharan Africa (8).

Nutrition-related factors, along with pneumonia, diarrhea, and complications of prematurity, contribute to the leading cause of death among under-five children in Ethiopia, accounting for 45%, 21%, 14%, and 12%, respectively (9). For children with SAM, the risk of death is approximately tenfold higher compared to well-nourished children. This deficiency can have long-lasting effects on child health and their overall development (10). SAM is both a medical and a social problem, and its management requires both medical and social interventions (11). SAM is also an economic burden for a country, which increases the direct and indirect costs of medical care, reduces workforce participation, increases dependency on social safety nets, and lowers overall productivity (12).

Severe acute malnutrition is a major public health problem in Ethiopia, where 7% of under-five children are affected by wasting and 1% of them are severely wasted (13). According to the 2016 Ethiopian Demographic and Health Survey (EDHS), the prevalence rate of stunting has significantly reduced from 58% to 38% in 2000 and 2016, respectively (14). According to recent literature, recovery rates for inpatient treatment of severe acute malnutrition using the WHO standard in Ethiopia range from 33.6% to 88.4% (15). The research findings highlighted several contributing factors to the high burden of severe acute malnutrition in Ethiopia, including sociodemographic characteristics, nutritional-related factors, medical comorbidity, and clinical, financial, and treatment-associated factors, which were identified as important predictors of recovery time (16, 17). Moreover, factors such as poverty, failure to breastfeed exclusively, maternal factors such as inadequate nutrition during pregnancy, failure to gain the appropriate weight, illnesses such as diarrhea and acute respiratory infections, poor consumption of vitamin supplements, large family size, poor sanitation, lower maternal education, low birth weight, lack of breastfeeding, and personal food preference were all considered causes and/or risk factors of malnutrition (18, 19).

Ethiopia has been implementing nationally modified WHO guidelines for the management of severe acute malnutrition and Seqota declarations as part of the Second National Nutrition Program (NNP-II), but the recovery rate from SAM is still far from expected (20). Previous studies in Ethiopia also reported that the recovery rate from SAM was reported ranging from 43.59% to 87% (17, 21), and most studies determined that the recovery rates are lower than the recommended WHO and national Sphere standards (22). Moreover, there is variability in the recovery time, rate, and predictors across previous studies. It is also crucial to understand the current context and the status of recovery from SAM during the period of COVID-19 and other outbreaks. Therefore, this study was conducted to determine the recovery status and its associated predictors among children with SAM who were admitted at Tirunesh Beijing Hospital (TBH) from July 2019 to June 2023.

## 2 Methods

### 2.1 Study area and period

The study was conducted at Tirunesh Beijing Hospital (TBH) in Addis Ababa, Ethiopia. TBH is a referral hospital that provides services for about 605,266 people per year. Severely malnourished children are directly admitted to the nutritional rehabilitation unit (a subsection of the pediatric ward). According to hospital records, over 18,000 under-five children visit the hospital every year.

### 2.2 Study design and population

An institutional-based retrospective follow-up study was conducted among all eligible cases of SAM aged 6–59 months who received treatment at the TBH nutritional rehabilitation unit (NRU) between July 2019 and July 2023.

### 2.3 Eligibility criteria

All cases of children with SAM treated in the TBH nutritional rehabilitation unit (NRU) between July 2019 and June 2023 were included in the study. However, those SAM cases whose card was incomplete or who were admitted for other causes were excluded from the study.

### 2.4 Sample size determination and sampling procedure

The sample size was calculated using a double population proportion formula and assumptions of 80% power, a 95% confidence interval, and a marginal error of 5%.

Finally, by taking the maximum sample size among the above-listed, the final sample size required was 461. The total number of admitted and treated children in TBH for SAM from July 2019 to June 2023 was 501. We included 461 medical records of children after excluding 40 incomplete records (Table 1).

**Table 1:** Sample size determination.

Variables	% of unexposed with outcome	RR	Power	Ratio	Sample size	Incomplete charts (10%)	Final sample	Reference
Health facility	57.1%	0.52	80%	2	362	36	398	(40)
Diarrhea	57.8	1.9	80%	2	419	42	461	(41)

RR, relative risk.

### 2.5 Study variables and operational definition

Time to recovery was the outcome variable in this study, and the independent variables were categorized as follows.

#### 2.5.1 Sociodemographic factors

Age, sex of the child, place of residence, season of admission, and vaccination status.

#### 2.5.2 Anthropometry and type of SAM

Anthropometry at admission [weight, height, mid-upper-arm circumference (MUAC) for 6–59 months], weight gain, admission diagnosis (marasmus, kwashiorkor, and marasmic-kwashiorkor), and edema.

#### 2.5.3 Medical comorbidity

Pneumonia, HIV serostatus, diarrhea, hemoglobin level, malaria, TB, dehydration, consciousness, and shock.

#### 2.5.4 Medication and treatment

IV fluid intake, IV antibiotic treatment, blood transfusion, routine medication (amoxicillin, ampicillin, gentamicin, etc.), and special medication.

#### 2.5.5 Nutritional therapy-related factors

Nasogastric (NG) tube use, plumpy nuts, F-100 and F-75 intake, and play stimulation.

### 2.6 Operational definition

#### 2.6.1 Protein calorie malnutrition

A state of inadequate food intake that takes place in the absence of a substantial injury, inflammation, or other disease that triggers a systemic inflammatory response (23).

#### 2.6.2 Chronic malnutrition

Defined using height for age (HFA), as *Z*-scores between −3 and −2 for moderate chronic malnutrition and *Z*-scores less than −3 for severe chronic malnutrition.

#### 2.6.3 Time to recovery

Determined by calculating the difference (in days) from the start of treatment until the child is discharged due to recovery.

#### 2.6.7 Severe acute malnutrition (SAM)

Defined by a very low weight for height (WFH)/weight for length, clinical signs of bilateral pitting edema, or a very low mid-upper arm circumference.

#### 2.6.8 Recovered (cured)

Defined when the child reaches ≥85% of the median weight for height (WFH) or WFH *Z*-score of greater than or equal to −2 on more than one occasion or no edema for 10 days (23).

#### 2.6.9 Censored

Those SAM children who were defaulted, transferred, or dead were considered censored observations.

#### 2.6.10 Defaulted

A child who was not seen for at least 2 weeks after starting treatment and confirmed by a home visit (23).

#### 2.6.11 Appetite test

Assessed by offering the child ready-to-use therapeutic food (RUTF). If the child passes the appetite test and has no other complications, then the child is considered for rehabilitation at home if not treated in the hospital (24).

#### 2.6.12 Death

Refers to a child’s death and has to be confirmed by a home visit.

### 2.7 The context of acute malnutrition services in Ethiopia

**Figure 1:**
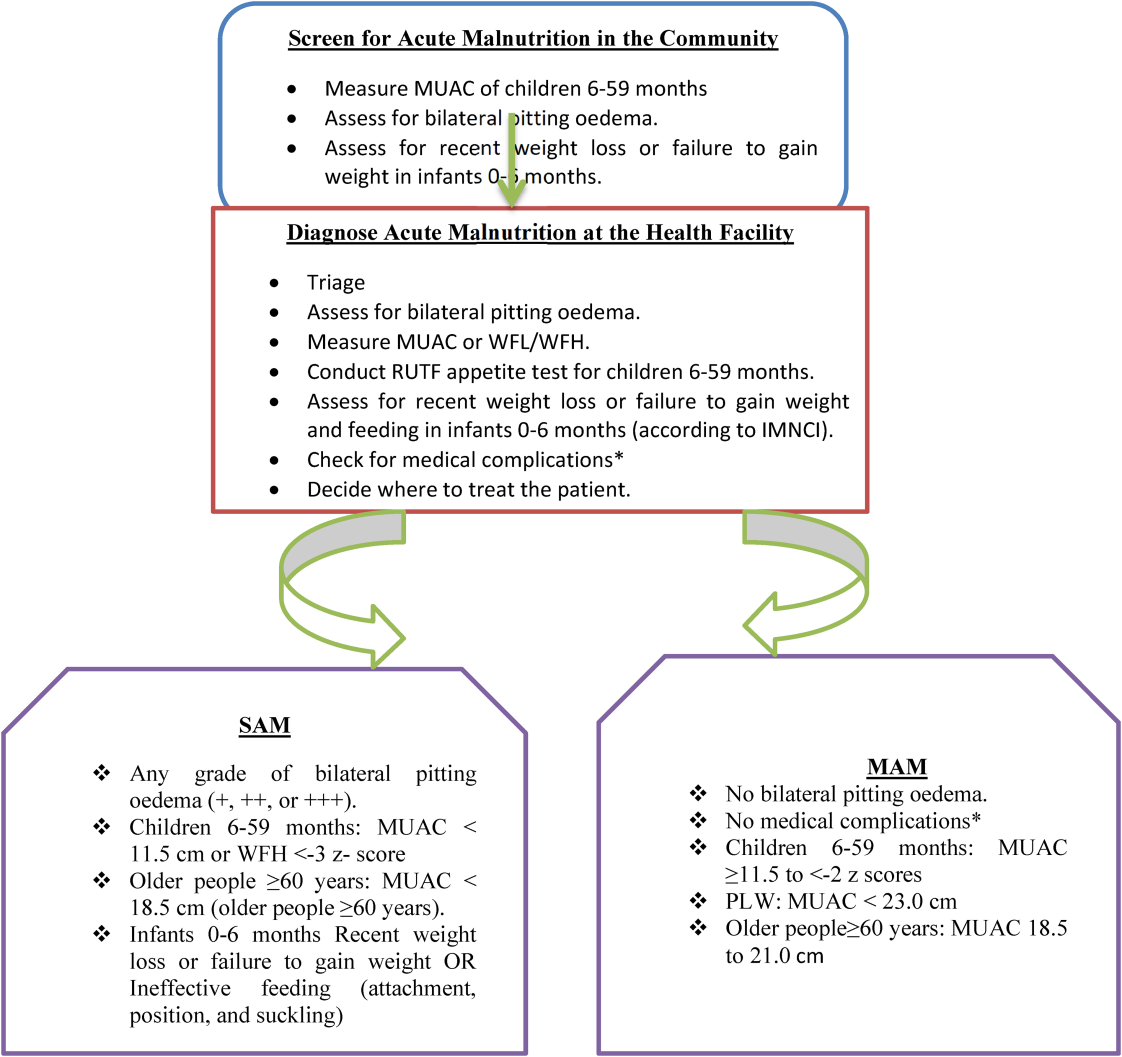
A flowchart adapted from the National Guideline for Management of Acute Malnutrition in Ethiopia.

The care and treatment for severe acute malnutrition (SAM) and moderate acute malnutrition (MAM) are integrated into the routine healthcare system. In line with the National Nutrition Program (NNP) II 2016–2020 and the Health Sector Transformation Plan (HSTP) 2015/16–2019/20, services for the management of SAM and MAM are delivered through the health system including health posts (HP), health centers (HC), and zonal, regional, and referral hospitals. The service is provided using a flow chart (Figure 1).

### 2.8 Data collection and quality control

Data were collected using a structured data abstraction format, and it was abstracted from SAM registries and cards of children retrieved from the card room using medical record numbers. Three graduated diploma nurses were recruited to collect the data from the patient's medical records and SAM treatment registry. One supervisor and the principal investigator followed the data collection closely. The length of stay and average weight gain were calculated from the available recorded secondary information. Anthropometric data of patients and other data were taken from their medical cards and the SAM registry. All records of 6–59-month-old children with SAM who were admitted to the stabilization center of TBH from July 2019 to June 2023 were included in this study. However, incomplete records that missed sociodemographic information, comorbidities, routine medications, and patient treatment outcomes (i.e., death, recovery, transfer out, and default) were excluded.

The validity of the questionnaire was assessed by subject-matter experts and senior researchers. In addition, the reliability of the tool was checked using Cronbach’s alpha coefficient, and the result was 0.73, meaning that the assumption was not violated. Moreover, to assure data quality, the data collection tool was pretested on 5% of the total samples at Zewditu Memorial Hospital. After the pretest, necessary amendments to the tool were made for the final data collection. A one-day training was given to the data collectors on how to extract the data from the patient registry.

### 2.9 Data processing and analysis

Data were coded and entered into EpiData software. Then it was exported to SPSS version 26 for further analysis. The age, weight, height, and edema were further exported to the ENA for SMART software to calculate the WFH% and HFA *Z*-score from admission and discharge height and weight measurements. A survival curve was used to display the survival (time to cure) among different characteristics. The outcome variable was dichotomized as recovered and censored for survival analysis. The Kaplan–Meier test was done to compare the median survival time among different groups. Factors related to time to recovery were analyzed using a multivariable Cox proportional hazard regression model. A crude and adjusted hazard ratio (aHR) with a 95% confidence interval was determined. To control the confounding variables, multivariable Cox proportional hazard regression was used. Variables with a *p*-value of <0.25 in bivariate Cox regression analysis were selected and included in multivariable Cox regression analysis. Finally, a *p*-value of <0.05 was used to declare a statistically significant association.

### 2.10 Ethical consideration

Ethical clearance was obtained from the Research and Ethical Review Committee of the Addis Ababa Medical and Business College. Then a supporting letter was written to the administrative body of TBGH. The information collected was kept confidential. Written informed consent was obtained from the hospital manager for accessing the medical records of SAM patients.

## 3 Results

### 3.1 Sociodemographic characteristics

This study was conducted with a total of 461 study participants, which yields a response rate of 100%. The minimum and maximum ages of the children were 6 and 59 months, respectively, with a mean and standard deviation of 17 ± 12 months. Among them, 166 (36%) were in the age category of 6–11 months, and 170 (36.9%) were in the age category of 12–23 months. Moreover, 232 (50.3%) of the study participants were female, and 449 (96.7%) were urban residents (Table 2).

**Table 2:** Sociodemographic characteristics of the study participants.

Variables	Category	Frequency (*n*)	Percentage (%)
Age (in months)	6–11	166	36.0
	12–23	170	36.9
	24–35	62	13.4
	36–47	24	5.2
	48–59	39	8.5
Sex	Male	229	49.7
	Female	232	50.3
Place of residence	Urban	446	96.7
	Rural	15	3.3

### 3.2 Medication and nutritional supplementation-related characteristics

Among the study participants, 185 (40.1%) had edema during admission, and 429 (93.1%) had complications during admission. Nearly half (232, 50.3%) had diarrhea at admission and 46 (10%) were diagnosed with TB infection during admission. Similarly, 28 (6.1) had HIV infection at admission, and the majority 192 (41.6%) had pneumonia at the time of admission. A total of 223 (48.4%) were fully vaccinated, and only 180 (39%) were exclusive breastfeeding. Almost all of the study participants (457, 99.1%) were used the routine medication and nutritional therapy. Moreover, 299 (64.9%) of the children received vitamin A supplementation, and 222 (48.2%) received amoxicillin supplementation. Only 1.1% of children received folic acid during admission (Table 3).

**Table 3:** Medication and nutritional supplementation-related characteristics.

Variables	Category	Frequency (*n*)	Percentage (%)
Edema at admission	Yes	185	40.1
	No	276	59.9
Complication at admission	Yes	429	93.1
	No	32	6.9
Diarrhea at admission	Yes	232	50.3
	No	229	49.7
TB infection at admission	Yes	46	10
	No	415	90.0
HIV infection at admission	Yes	28	6.1
	No	433	93.9
Pneumonia at admission	Yes	192	41.6
	No	269	58.4
URTI at admission	Yes	101	21.9
	No	360	78.1
Anemia at admission	Yes	89	19.3
	No	372	80.7
Sepsis at admission	Yes	12	2.6
	No	449	97.4
Malaria at admission	Yes	25	5.4
	No	436	94.6
Dehydration at admission	Yes	130	28.2
	No	331	71.8
Shock at admission	Yes	107	23.2
	No	354	76.8
Vaccination status	Not vaccinated	97	21.0
	Partially vaccinated	141	30.6
	Fully vaccinated	223	48.4
Exclusive breastfeeding	Yes	180	39.0
	No	281	61.0
Vitamin A supplementation	Yes	299	64.9
	No	162	35.1
Amoxicillin provision	Yes	222	48.2
	No	239	51.8
Gentamycin provision	Yes	297	64.4
	No	164	35.6
Deworming	Yes	177	38.4
	No	284	61.6
Blood transfusion at admission	Yes	33	7.2
	No	428	92.8

URTI, upper respiratory tract infection.

### 3.3 Nutritional assessment and intervention-related characteristics

Most of the children (315, 68.3%) failed the appetite test. Concerning the weight for height of children, 286 (62%) were lower than 70%, and 121 (26.2%) were between 70% and 80%. Similarly, 175 (38%) of children had a height for age more than 95%, and 156 (33.8%) had 90%–95% (Table 4).

**Table 4:** Nutritional assessment and intervention-related characteristics.

Variables	Category	Frequency (*n*)	Percentage (%)
Appetite test	Failed	315	68.3
	Passed	146	31.7
WFH	<70%	286	62.0
	70%–80%	121	26.2
	>80%	54	11.8
HFA	>95%	175	38.0
	90%–95%	156	33.8
	85%–90%	66	14.3
	<85%	64	13.9
MUAC	<115 mm	347	75.3
	115 mm and above	114	24.7
Plumpy nut provision	Yes	172	37.3
	No	289	62.7
F100 provision	Yes	273	59.2
	No	188	40.8
F75 provision	Yes	454	98.5
	No	7	1.5
Play stimulation	Yes	395	85.7
	No	66	14.3

MUAC, mid-upper-arm circumference; WFH, weight for height. HFA, height for age.

### 3.4 Survival time to recovery from SAM

**Figure 2:**
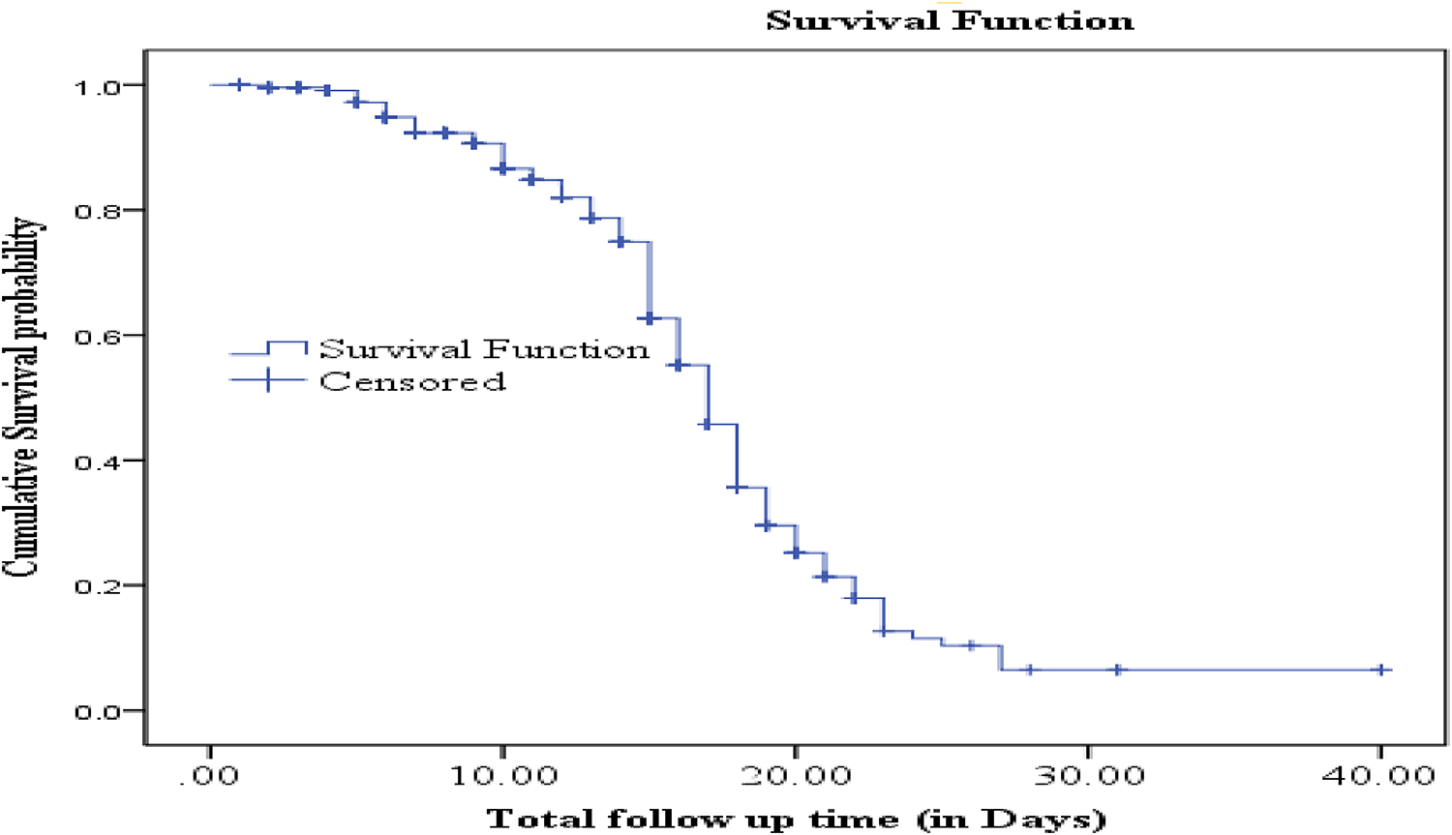
The Kaplan–Meier estimate of the survival time to recovery.

The cumulative proportion of people surviving at the end of the 2nd, 15th, and 27th days were 99.6%, 62.7%, and 6.5%, respectively. The median survival time to recovery was 17 days (95% CI: 16.39–17.60) (Figure 2).

### 3.5 Incidence rate of recovery from SAM

**Figure 3:**
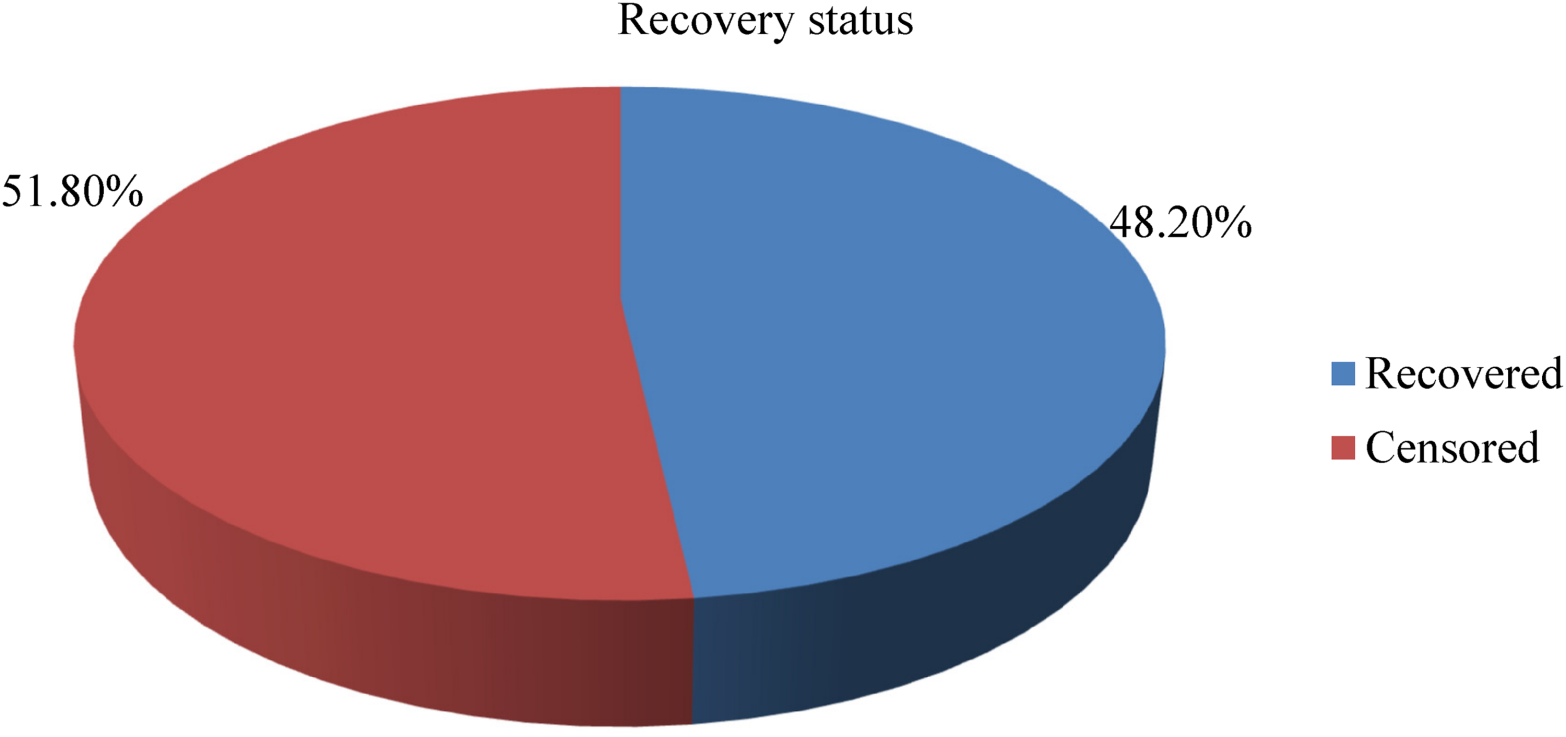
Recovery status of the study participants.

In this study, a total of 461 children were followed for a minimum of 1 day and a maximum of 40 days, with an overall median length of stay of 13 days ± 6 days. Based on this, the total children's time of observation was 5,878 child days. Furthermore, the incidence rate of recovery from SAM among 6–59-month-olds with SAM was 37.8 per 1,000 child days’ observation, and the percentage of study participants recovered was 48.2% (Figure 3).

### 3.6 Predictors of time to recovery

Predictors of time to recovery were assessed initially using bivariable Cox proportional hazard regression analysis with a *p*-value of <0.25 to identify factors that potentially affect the time to recovery. As a result, sex, residence, exclusive breastfeeding, vaccination status, type of SAM, presence of edema, complication at admission, diarrhea, TB, HIV, pneumonia, upper respiratory tract infection (URTI), amoxicillin, ampicillin, gentamicin, deworming, special medication, IV antibiotic, NG tube use, and play stimulation were identified as a candidate variables for the final model (*p* < 0.25).

Besides, multivariable Cox proportional hazard regression analysis was performed to select the predictor variables by adjusting for confounders. As a result, only exclusive breastfeeding, complications at admission, diarrhea, deworming, and amoxicillin provision during admission were found to be independent predictors of recovery status.

As indicated in the table below, controlling all other variables constant, children who received only breast milk had a 97% higher chance of recovery time compared to their counterparts (aHR = 1.97, 95% CI: 1.45–2.68). Similarly, children who received amoxicillin during admission have a 62% lower risk of delayed recovery time as compared with those who did not receive amoxicillin during admission (aHR = 1.62, 95% CI: 1.11–2.35). Moreover, the recovery time was more than two times better among children who were dewormed as compared with their counterparts (aHR = 2.14, 95% CI: 1.48–3.99).

On the other hand, SAM children with complications at admission had a 59% (aHR = 0.41, 95% CI: 0.23–0.73) higher risk of delayed recovery time as compared to those children without complications at admission, and children who have diarrhea during admission had a 36% (aHR = 0.64, 95% CI: 0.45–0.91) higher risk of delayed recovery time than their counterparts (Table 5).

**Table 5:** Cox proportional hazard regression analysis for predictors of time to recovery.

Variable	Category	Outcome status		cHR (95% CI)	aHR (95% CI)
		Recovered	Censored		
Residence	Urban	219	227	2.22 (0.71–6.93)	1.99 (0.62–6.43)
	Rural	3	12	1	1
Exclusive breastfeeding	Yes	120	60	1.57 (1.21–2.05)	1.97 (1.45–2.68)**
	No	102	179	1	1
Vaccination	Not vaccinated	23	74	0.52 (0.34–0.82)	0.98 (0.60–1.60)
	Partially vaccinated	77	64	0.68 (0.51–0.91)	0.91 (0.64–1.29)
	Fully vaccinated	122	101	1	1
Presence of edema	Yes	66	119	0.67 (0.50–0.89)	0.68 (0.44–1.05)
	No	156	120	1	1
Complication at admission	Yes	202	227	0.33 (0.21–0.53)	0.41 (0.23–0.73)**
	No	20	12	1	1
Diarrhea	Yes	56	176	0.39 (0.29–0.54)	0.64 (0.45–0.91)**
	No	166	63	1	1
TB	Yes	10	36	0.49 (0.26–0.92)	0.51 (0.24–1.07)
	No	212	203	1	1
HIV	Yes	5	23	0.37 (0.15–0.89)	1.28 (0.45–3.66)
	No	217	216	1	1
URTI	Yes	55	46	1.25 (0.92–1.70)	1.33 (0.94–1.89)
	No	167	193	1	1
Amoxicillin	Yes	146	76	2.14 (1.62–2.82)	1.62 (1.11–2.35)**
	No	76	163	1	1
Gentamicin	Yes	137	160	0.77 (0.59–1.01)	0.92 (0.64–1.34)
	No	85	79	1	1
Deworming	Yes	122	55	2.30 (1.77–3.00)	2.14 (1.48–3.09)**
	No	100	184	1	1
IV antibiotic	Yes	189	227	0.56 (0.39–0.82)	1.18 (0.73–1.90)
	No	33	12	1	1
Play stimulation	Yes	220	175	5.11 (1.26–20.69)	3.02 (0.69–13.19)
	No	2	64	1	1

cHR, crude hazard ratio; aHR, adjusted hazard ratio; URTI, upper respiratory tract infection.

* Significant variables at *p* < 0.05. **Significant variables at a *p* < 0.001.

## 4 Discussion

The current study revealed a recovery rate of 48.2%, which was low when compared to the sphere standards that recommend the cure rate should exceed 75% (25) in malnourished children on relevant treatment protocols. This low recovery rate may be attributable to a late presentation (3), a higher percentage stabilized and transferred out to Outpatient Therapeutic Feeding Program (OTP) (25.2%), and patient overload (11). As well, this low recovery rate may be attributable to non-adhering to the standard protocol for the management of SAM (26). Thus, to achieve a better cure rate, the management of the SAM standard protocol needs to be implemented properly. Lastly, in this study, the low recovery rate may be due to mismanagement of children, such as partial prescription of routine medication, or comorbidity at admission, such as the presence of diarrhea.

This study also exposed important information about the nutritional recovery time of children with SAM managed in a nutritional rehabilitation unit and predictors of time to nutritional recovery. The median time of recovery from SAM was 17 days (95% CI: 16.39–17.6). This is in line with a study finding from St. Paul's Hospital Millennium Medical College, Ethiopia (27). The median time of recovery in this study was higher than findings reported from different parts of Ethiopia (3, 18, 26, 28), but it was lower than some other studies done at Karat and Fasha stabilization centers in southern Ethiopia (26 days) and Shebedino district in southern Ethiopia (36 days) (29, 30). These variations could be due to differences in the quality of care, differences in the qualifications of care providers, and differences in the settings of stabilization centers. The late detection of SAM and the late referral to the stabilization centers might also contribute to the differences in the times of recovery.

Exclusive breastfeeding was found to be a predictor of time to recovery from SAM among children aged 6–59 months. Children who receive exclusive breastfeeding have 1.97 times better recovery times as compared with their counterparts (aHR = 1.97, 95% CI: 1.45–2.68). The reason behind this might be that exclusive breastfeeding might help to prevent undernutrition and infection and might speed up recovery after SAM and associated diseases (31).

A non-occurrence of complications at admission was identified as a predictor of time to recovery from SAM. Recovery time was delayed by 59% among children who had complications at admission as compared with their peers (aHR = 0.41, 95% CI: 0.23–0.73). This finding was consistent with a study conducted in northwestern Ethiopia, which reported that children who had anemia on admission had a lower chance of early recovery (26). This indicates that children with complications of anemia can not feed themselves and might be at risk for poor recovery and need attention during treatment. Similarly, the findings of complications at admission were consistent with the study done in southern Ethiopia, which revealed SAM children who did not develop complications at Therapeutic Feeding Centers (TFCs) had a significantly higher nutritional recovery rate than their counterparts (29). The reason behind this could be that the presence of comorbid conditions characterized by inadequate food intake may lead to a fast depletion of nutrients and delayed nutritional recovery.

A non-occurrence of diarrhea during admission was identified as a predictor of time to recovery from SAM. The recovery rate was delayed by 36% among children who had diarrhea during admission as compared with their counterparts (aHR = 0.64, 95%CI: 0.45–0.91). This finding was consistent with a study report from Tigray, Northern Ethiopia, which reported that children with no diarrhea had a more than two times higher probability of recovering from SAM (32, 33). The possible explanation could be that diarrhea might cause increased peristalsis movement of food in the gastrointestinal tract and might decrease the rate of absorption, which is exacerbated by nutrition. In addition, children with diarrhea commonly have a loss of appetite.

Amoxicillin provision during admission had a positive association with a rapid time to recovery from SAM. Children who received amoxicillin during admission have 1.62 times better recovery as compared with those who did not receive amoxicillin during admission (aHR = 1.62, 95% CI: 1.11–2.35). This result was supported by studies from Northwest Ethiopia (34), Eastern Ethiopia (35), and Lilongwe, Malawi (36). This can be explained by the supportive effect of amoxicillin in the treatment of infections and other complications associated with SAM (4),

which might be related to amoxicillin specifically reducing the risk of transfers to inpatient care for clinical complications due to gastroenteritis. In addition, the poor mucosal integrity in malnourished children allows the translocation of bacteria across compromised intestinal surfaces, resulting in bacteremia (37, 38).

Deworming was found to be a predictor of time to recovery from SAM. The recovery rate was more than two times better among children who were dewormed as compared with those who were not dewormed (aHR = 2.14, 95% CI: 1.48–3.09). This finding was consistent with a study conducted in the northern region and southwest Ethiopia (31, 39). Due to the unhygienic environment, many children in the developing world have diverse types of intestinal parasites; thus, children aged 2 years and older are recommended to be dewormed every 6 months (40). The worm affects the patient's appetite due to its abdominal destination and causes anemia, which might further decrease the rate of recovery. In addition, this might be because children with SAM are at an increased risk of infection and intestinal inflammation (31), which could impair nutrient absorption and delay the recovery time.

### 4.1 Strengths and limitations of the study

The main strengths of this study might be related to the study design employed, which might assess the cause-and-effect relationship. Although this study is not free from limitations, as it was based on secondary data obtained from patient medical records, it lacks the incorporation of some variables like maternal educational status, family size, wealth index, and food security. Furthermore, potential bias due to incomplete records was possible. Thus, the findings of this study should be interpreted in consideration of these limitations.

## 5 Conclusion and recommendation

The time to recovery among the current study participants was low compared with the Sphere standard (<4 weeks) (24). Moreover, exclusive breastfeeding, complications at admission, diarrhea, amoxicillin provision at admission, and deworming were independent predictors. Therefore, appropriate provision of routine medication and early management of medical comorbidity as per the national SAM management protocol can reduce the mortality of children with severe acute malnutrition significantly.

## Data Availability

The original contributions presented in the study are included in the article/Supplementary Material; further inquiries can be directed to the corresponding author.
